# CONTEMPORARY CORE JOB FUNCTIONS OF NURSING HOME SOCIAL SERVICES DEPARTMENT IN THE UNITED STATES

**DOI:** 10.1093/geroni/igz038.3408

**Published:** 2019-11-08

**Authors:** Amy L Lemke, Mercedes Bern-Klug

**Affiliations:** 1 University of Iowa School of Social Work, Iowa City, Iowa, United States; 2 University of Iowa, Iowa City, Iowa, United States

## Abstract

Nursing home social services departments (NH SS-Ds) are involved in a myriad of duties i.e., from care manager, patient advocate to counselor. But it is unclear how much variation there is across departments. Begging the question, what are the most common core functions of NH SS-Ds in the United States? A nationally representative cross-sectional sample of 922 NH SS-Ds completed a survey on-line or via mail. They were asked to indicate the extent their department was involved in 46 job functions. The listing of functions was inspired by the literature and pilot tested. The most common core functions would include those whereby at least 2/3s of the respondents would report “always/usually” being involved. 32 of the 46 functions were rated as “always/usually” and therefore qualified as the most common core functions. The five most common were discussing with staff the discharges of long-term as well as short-stay residents; arranging services for residents returning to the community; creating care plans; and mediating issues between residents. There were only two items whereby less than 10% of departments were “always/usually” involved – working with volunteers and helping feed residents. This survey tool successfully represented core SS-D functions as remarkable similarity across SS-Ds in the US existed. Responsibilities associated with care planning and care transitions were heavily featured, thus reflecting recent federal guidelines changes. The study’s results are pertinent to researchers, educators as well as nursing home administrators for role clarification and identification of training domains.

